# Platelet membrane biomimetic nanoparticle-based targeted delivery system of simvastatin for the treatment of ischemic stroke

**DOI:** 10.1371/journal.pone.0354184

**Published:** 2026-07-23

**Authors:** Qin Li, Rongyuan Li, Lu Lin, Meiting Gong, Yu Liang, Daming Sui

**Affiliations:** 1 Department of Anesthesiology, Xindu District People’s Hospital of Chengdu, Sichuan, Chengdu, China; 2 Department of Pain Medicine, The General Hospital of Western Theater Command, Sichuan, Chengdu, China; 3 Department of Anesthesiology, The General Hospital of Western Theater Command, Sichuan, Chengdu, China; 4 Medical College of Southwest Jiaotong University, Sichuan, Chengdu, China; Mass Eye Infirmary, Harvard Medical School / Northeastern University, UNITED STATES OF AMERICA

## Abstract

Blood–brain barrier (BBB) disruption and excessive neuroinflammation are pivotal drivers of cerebral ischemia-reperfusion injury. Although simvastatin (SV) possesses potent pleiotropic effects in promoting BBB repair and attenuating inflammation, its clinical translation for ischemic stroke is severely hampered by poor BBB penetration, low lesion accumulation, and the need for high systemic doses that increase the risk of off-target toxicity (e.g., myopathy). To address these barriers, herein, we developed a platelet membrane–biomimetic nanoparticle system (pmPLGA@SV) designed to leverage the innate affinity of platelets for injured vasculature for lesion-targeted SV delivery at a lower effective dose. Physicochemical characterization confirmed the successful cloaking of platelet membranes onto SV-loaded PLGA cores. In vitro, pmPLGA@SV demonstrated superior therapeutic versatility: it effectively scavenged reactive oxygen species in oxygen glucose deprivation/reoxygenation-treated PC12 cells and orchestrated microglial repolarization from a pro-inflammatory M1 phenotype toward an anti-inflammatory M2 state in BV2 cells, significantly modulating the secretion of IL-1β and IL-10. In vivo, pmPLGA@SV treatment dramatically reduced the cerebral infarct volume, exhibiting significant superiority over free SV. Furthermore, longitudinal behavioral assessments over 21 days demonstrated that pmPLGA@SV markedly accelerated motor and sensory-motor functional recovery, accompanied by consistent body weight regain and improved neurological scores. Mechanistically, pmPLGA@SV facilitates a synergistic therapeutic approach by mitigating neuronal oxidative stress and remodeling the inflammatory microenvironment. This study demonstrates that pmPLGA@SV serves as a robust biomimetic platform for the integrated treatment of neurovascular unit damage, offering a promising strategy for long-term neuroprotection and functional rehabilitation following ischemic stroke.

## Introduction

IIschemic stroke is a major cause of global mortality and long-term disability worldwide. It is characterized by the sudden interruption of blood flow to the brain, resulting in neural tissue death [1,2]. Although timely reperfusion through pharmacological thrombolysis or mechanical thrombectomy is the clinical gold standard, its application is severely restricted by a narrow therapeutic window and the risk of ischemia–reperfusion injury [3,4]. Accumulating evidence suggests that the primary ischemic insult is rapidly followed by a complex cascade of secondary injuries, including blood–brain barrier (BBB) disruption and uncontrolled neuroinflammation, which significantly exacerbate neurological deficits and impede functional recovery [5].

The neurovascular unit (NVU), comprising neurons, endothelial cells, and glial cells, plays a critical role in maintaining brain homeostasis [6,7]. Post-stroke, the overproduction of reactive oxygen species (ROS) instigates significant oxidative damage to neurons and exacerbates the subsequent neuroinflammatory cascade [8,9]. Concurrently, microglia, the resident immune cells of the brain, are activated and polarized into distinct phenotypes: the pro-inflammatory M1 phenotype, which secretes deleterious cytokines like IL-1β, and the anti-inflammatory M2 phenotype, which promotes tissue repair and neuroprotection [10,11]. Consequently, modulating microglial polarization from M1 to M2 and mitigating neuronal oxidative stress have emerged as promising synergistic strategies for stroke therapy [12,13].

Simvastatin (SV), a widely used HMG-CoA reductase inhibitor, has gained significant attention for its potent pleiotropic effects beyond cholesterol lowering [14–17]. Recent studies have highlighted its capacity to attenuate oxidative stress, stabilize the BBB, and orchestrate immunomodulatory responses in various neurological disorders [18,19]. However, the clinical translation of SV for the treatment of acute ischemic stroke is severely hampered by its suboptimal pharmacokinetic profile, which includes low aqueous solubility, rapid systemic clearance, and particularly poor penetration across the BBB [20,21]. In order to achieve the requisite therapeutic concentrations within the ischemic penumbra, high systemic doses are frequently required. However, this inevitably increases the risk of side effects, including myopathy and hepatic dysfunction [22,23]. These issues highlight the urgent need for a targeted delivery strategy that can concentrate SV at the ischemic lesion while reducing systemic exposure [20,21].

To overcome these delivery barriers—specifically the poor BBB penetration and insufficient lesion accumulation that necessitate high systemic doses of SV—nanotechnology-based platforms have been developed to enhance drug stability and targeting [24,25]. Among these, biomimetic camouflaging—specifically the use of platelet membranes (pm)—represents a paradigm shift in drug delivery [26,27]. Platelets possess an inherent biological tendency to adhere to injured vascular endothelium and inflammatory sites, a property that is governed by specific surface receptors [28,29]. By cloaking SV-loaded Poly lactic-co-glycolic acid (PLGA) nanoparticles with platelet membranes (pmPLGA@SV), it is possible to bypass immune surveillance, prolong systemic circulation, and achieve lesion-specific drug accumulation through active homing mechanisms, thereby maximizing therapeutic efficacy while minimizing off-target toxicity [30,31]. Consequently, this biomimetic platform allows SV to exert its pleiotropic effects at a substantially lower effective dose, circumventing the high-dose toxicity that has limited its clinical application in acute stroke.

In the present study, we developed a platelet membrane–biomimetic nanoparticle system (pmPLGA@SV) for the targeted treatment of ischemic stroke. We hypothesized that this biomimetic platform could effectively deliver SV to the ischemic brain, simultaneously mitigating oxidative stress and remodeling the inflammatory microenvironment. In vitro, we investigated the ROS-scavenging capability of pmPLGA@SV in oxygen glucose deprivation/reperfusion (OGD/R)-treated PC12 cells, as well as its impact on M1/M2 polarization in BV2 microglia. In vivo, using a mouse model of transient middle cerebral artery occlusion (tMCAO), we comprehensively evaluated its effects on infarct volume reduction and, critically, on long-term neurological functional recovery. This study provides a robust strategy for integrating biomimetic nanotechnology with pleiotropic drug therapy to promote neurovascular restoration and functional rehabilitation.

## Materials and methods

### Materials

PLGA (75:25 MW = 15000) was purchased from Jinan Daigang Biomaterial Co., Ltd. (Jinan, China). Acetone was purchased from Adamas Reagent, Ltd. (Shanghai, China). Reagents for western blot analysis, including RIPA lysis buffer, protein loading buffer, and TBST (Tris-buffered saline with Tween® 20) were purchased from GenScript Biotech Corporation (China).

### Cell lines and culture conditions

Mouse microglia BV2 cells, mouse brain capillary endothelial bEnd. 3 cells, and PC12 paraganglioma cell lines were obtained from iCell Bioscience Inc (Shanghai, China). BV2 and bEnd.3 were cultured in DMEM (Gibco) containing 10% fetal bovine serum (FBS) (ExCell Bio) at 37℃ with 5% CO2. PC12 cells were cultured in 1640 medium (Gibco) with 10% FBS.

### Animals

C57BL/6J mice (25 ~ 30 g, male) were purchased from Gempharmatech Co., Ltd. (Nanjin, China; license: SCXK (Chuan) 2020−034) were used in the experiments. All animals were maintained under 12 h light-dark cycle at 25 ± 1℃ and 50% ~ 60% humidity with access to food and water ad libitum. All experiments strictly followed the 3R principles for the use of experimental animals and provision of humane care. The method of euthanasia was performed in accordance with the AVMA Guidelines for the Euthanasia of Animals (2020 edition) and is detailed in the Tissue collection and sacrifice subsection below.

### Characterization of pmPLGA@SV

PLGA nanoparticles were prepared by solvent evaporation method. 50 mg PLGA were dissolved in 5 mL acetone, added dropwise into 50 mL water, and stirred for 4 h at room temperature. The spherical nanoparticles could be obtained with the evaporation of acetone. For simvastatin-loaded nanoparticles, the drug and PLGA were dissolved in acetone at a weight ratio of 1:10 (SV: PLGA). The solution was then added dropwise into water under stirring. The resulting nanoparticle suspension was dialyzed against 1 L of distilled water using a dialysis membrane with a molecular weight cutoff (MWCO) of 3.5 kDa at 4℃ for 24 h, with water changed every 6 h, to remove unloaded simvastatin and residual acetone.

To prepare platelet membrane-coated nanoparticles, 1 mL of mouse whole blood was first centrifuged (1,500 × g, 15 min) to isolate platelet-rich plasma. Platelets were then collected and lysed via probe sonication (4℃, 10 × 30 s pulses) to yield membrane vesicles. The vesicles were resuspended in 1 mL of PBS and combined with 1 mL of PLGA suspension (1 mg/mL). The mixture was subsequently subjected to co-extrusion through 200 nm polycarbonate membranes for 10 cycles to obtain pmPLGA nanoparticles. pmPLGA@SV was prepared following a similar protocol.

The particle size, PDI and zeta potential of the nanoparticles were determined by dynamic light scattering (DLS) (Zeta-Sizer, Malvern Nano-ZS90, Malvern, Ltd., UK). The morphology of nanoparticles was examined by transmission electron microscopy (TEM, TECNAI G2F20FEI, USA). The drug loading content (LC%) and entrapment efficiency (EE%) of the nanoparticles were detected by UV-vis (UV-2550 Shimadzu，Japan). DL and EE were calculated by the following formula:


$$\begin{array}{c} \text{LC }(\%)\;=\text{ m}/\text{mA }\times \;\text{1}\text{0}\text{0}\% \end{array}$$ (1)

$$\begin{array}{c} \text{EE }(\%)\;=\text{ m}/\text{mR }\times \;\text{1}\text{0}\text{0}\% \end{array}$$ (2)

Where m is the mass (mg) of simvastatin encapsulated in the nanoparticles, mA is the total mass of the drug-loaded nanoparticles, and mR is the initial mass (mg) of simvastatin used in the preparation.

**Zeta potential measurement:** Zeta potential of PLGA@SV and pmPLGA@SV was measured at 25℃ using a Zetasizer (Malvern Nano-ZS90) after dilution in PBS (pH 7.4). Three independent batches were measured, each in triplicate.

**Stability assessment:** Freshly prepared pmPLGA@SV was stored as a sterile suspension in PBS (pH 7.4) at 4℃. Aliquots were taken at 0, 1, 3, 7, 14 d to measure encapsulation efficiency (EE%) and hydrodynamic diameter.

**In vitro release study:** One milliliter of pmPLGA@SV suspension (1 mg/mL) was dialyzed against 10 mL of PBS (pH 7.4, 0.1% Tween 80) at 37℃ with gentle shaking (100 rpm). At predetermined time points (0, 6, 12, 24, 48, 72 h), 1 mL of release medium was withdrawn and replaced with fresh PBS. SV concentration in the withdrawn samples was quantified by UV-vis. The cumulative release percentage was calculated. All measurements were performed in triplicate.

### Characterization of platelet membrane coating

To confirm the successful coating of platelet membrane proteins on nanoparticles, gel electrophoresis and western blot were performed. The nanoparticle solution was centrifuged (12,000 × g, 15 min) and lysed with RIPA buffer, and the supernatant containing membrane proteins was collected after a second centrifugation. Protein concentrations were quantified by BCA assay and adjusted to 1 μg/μL. After denaturation, samples were separated on a 12% polyacrylamide gel (80 V for 30 min, then 120 V for 45 min). For Coomassie Blue staining, gels were destained until clear. For western blot, proteins were transferred to a PVDF membrane at 400 mA for 20 min, blocked with 5% skim milk, and sequentially incubated with primary antibody (overnight, 4℃) and secondary antibody (2 h, room temperature). Signals were detected by ECL chemiluminescence.

### Cell OGD/R model

The bEnd.3 cells or BV2 cells were cultured in incubator for 24 h of attachment. Then the culture medium was replaced with glucose-free and serum-free DMEM medium. After that, the cells were cultured in 95% N_2_ and 5% CO_2_ environment to simulate low oxygen condition. 3 h later, the medium was replaced with normal medium and the cells were transferred to normal incubator to simulate reperfusion injury.

### Cell phagocytosis experiment

The bEnd. 3 cells were seeded in 6-well plates (1 × 10^5^ cells/well) for 24 h of attachment. After OGD/R, cells were treated with rhodamine-loaded PLGA or pmPLGA for 2 h. Then the cells were washed and observed using fluorescence microscopy and flow cytometry.

### In vitro BBB model

The BBB model was established by seeding bEnd.3 cells onto Transwell inserts and monitoring integrity via TEER measurements. Upon successful model construction (TEER > 200 Ω/cm^2^), cells were subjected to OGD/R treatment. Subsequently, rhodamine-loaded PLGA or pmPLGA were added to the apical chamber. After 30 minutes, the fluorescence intensity in the basolateral chamber was quantified using a microplate reader to evaluate nanoparticle transport across the injured barrier.

### In vitro microglia polarization

PBS, PLGA@SV, and pmPLGA@SV were incubated with OGD/R treated BV2 cells for 24 h. Then, the cells were washed, digested to cell suspension, and labeled with CD206-FITC (1:100, eBioscience) and CD86-PE (1:100, Abcam) for flow cytometry (CytoFLEX, Beckman Coulter, USA) analysis. The gating strategy was as follows: live cells were gated on FSC-A/SSC-A, doublets were excluded using FSC-A/FSC-H and FSC-H/FSC-W, and then CD206 and CD86 expression were analyzed on the singlet population. The polarization ratio of BV2 cells were calculated according to the percentage of different phenotypes.

### ELISA

The levels of cytokines, including IL-1β, IL-10, were measured using commercial ELISA kits (Shanghai Enzyme-linked Biotechnology Co.). The supernatant was collected and centrifuged at 3000 rpm for 10 min. All supernatants were measured according to the manufacturer's instructions.

### In vitro ROS scavenging

PC12 (1 × 10^5^ cells per well) cells were seeded in 6-well plates. After cell attachment, cells were incubated under OGD condition for 2 h and in normal culture condition for 24 h. Then saline, PBS, PLGA@SV, and pmPLGA@SV were incubated with the cells. 6 h later, cells were washed, incubated with DCFH-DA (10 µM), and imaged by fluorescence microscopy.

### The tMCAO model

All animal procedures were approved by the Ethical Committee on Animal Testing at the General Hospital of Western Theater Command (Ethics Approval No. 2023ky119−1) and were performed in accordance with the ARRIVE guidelines.

Male C57BL/6J mice (25–30 g) were used. Anesthesia and analgesia: Anesthesia was induced with 5% isoflurane in 100% oxygen and maintained with 1.5–2% isoflurane in 100% oxygen via a nose cone throughout the surgery. The depth of anesthesia was confirmed by the absence of a pedal withdrawal reflex. For postoperative analgesia, buprenorphine (0.05 mg/kg, subcutaneously) was administered immediately after surgery and again at 12 hours post-surgery. Efforts to alleviate suffering: Body temperature was maintained at 37.0 ± 0.5℃ using a rectal probe and a feedback-controlled heating pad during surgery and until full recovery. After surgery, animals were placed in a recovery cage with a warming pad and monitored every 2 hours for the first 24 hours for signs of pain or distress (e.g., hunched posture, reduced locomotion, piloerection, decreased food/water intake). Any animal showing severe distress would have received additional buprenorphine (0.05 mg/kg, s.c.) or been humanely euthanized earlier; however, no animal met the early removal criteria in this study.

For tMCAO induction, the external carotid artery (ECA) and the internal carotid artery (ICA) on the right side were separated. Then, a specialized filament (wire clot) was inserted from the ECA into the ICA until the origin of the middle cerebral artery (MCA) was blocked. After 1 hour of ischemia, the filament was removed to allow reperfusion. Sham-operated mice underwent the same surgical procedure (anesthesia, neck incision, and carotid artery isolation) except that the filament was not inserted to induce occlusion. The Sham group served as a non-ischemic negative control.

Tissue collection and sacrifice: At the predetermined endpoint, mice were deeply anesthetized with 5% isoflurane in 100% oxygen. The chemical agent used for euthanasia was 5% isoflurane (inhalation overdose). After confirming the loss of pedal reflex, animals were decapitated, and the brains were rapidly removed. Decapitation was performed under deep isoflurane anesthesia to ensure that the animals did not experience pain or distress. Death was confirmed by cessation of heartbeat and respiration. Thus, the methods of sacrifice included an overdose of isoflurane as the primary chemical agent, followed by decapitation as a secondary physical method.

### TTC staining

Following humane euthanasia, all brain tissue collection was performed strictly in accordance with the 3R principles and the AVMA Guidelines for the Euthanasia of Animals to ensure ethical compliance. 2,3,5-Triphenyl-2H-Tetrazolium Chloride (TTC) staining was employed to evaluate cerebral infarction. Mouse brains were collected and sectioned, followed by incubation in 2% TTC solution at 37℃ for 30 min. Viable tissue stained crimson red due to the enzymatic reduction of TTC to formazan, whereas infarcted regions, devoid of dehydrogenase activity, remained pale white.

### Behavioral assessment

A series of behavioral tests were conducted to evaluate neurological recovery in mice. The modified neurological severity score (mNSS), has a total of 18 points, including 6 points for motor test, 2 points for sensory test, 6 points for the balance beam test, and 4 points for reflex loss and abnormal movement. For the rotarod test assessing motor coordination and endurance, mice were placed on a rotating rod that accelerated gradually from 5 to 20 rotations/min. Prior to formal testing, all mice underwent a 7-day training period to ensure they could remain on the rod for a maximum of 240 seconds. The mean duration spent on the rod was recorded as the latency to fall. For the cylinder test assessing forepaw use asymmetry, mice were placed in a transparent cylinder for a 10-min session, which was recorded using a video camera. Forepaw contacts against the cylinder wall were quantified as follows: L (number of left forepaw contacts), R (number of right forepaw contacts), and B (number of simultaneous contacts with both forepaws). The asymmetry rate was calculated using Equation (5).


$$\begin{array}{c} Asymmetry\;rate\;(\%)\;=\;(L-R)\;/\;(L\;+\;R\;+B)\;\times \;\text{1}\text{0}\text{0}\% \end{array}$$ (3)

For the adhesive test assessing the tactile response and sensorimotor function, a 5 × 5 mm^2^ patch was attached to the paralyzed forepaw of each mouse. The latency for the mouse to completely remove the patch was recorded as the removal time.

### Statistical analysis

Statistical analyses were performed using GraphPad Prism 10. For datasets satisfying assumptions of normality and homogeneity of variance, inter-group mean comparisons were performed using unpaired Student's *t*-test. Between-group differences were evaluated via one-way ANOVA. Quantitative results are expressed as mean ± SD, with all figure captions specifying the corresponding *p* values.

## Results

### Preparation and characterization of pmPELA@SV

**Fig 1:**
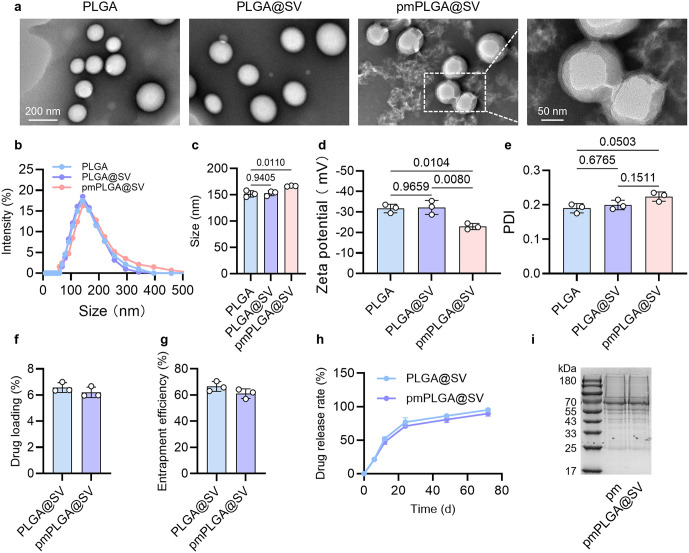
Physicochemical characterization of pmPLGA@SV. (a) Representative TEM images of PLGA, PLGA@SV, and pmPLGA@SV nanoparticles. (b) Hydrodynamic size distribution measured by DLS (representative of three independent batches). (c) Mean particle size of different nanoparticle formulations. (d) Zeta potential of PLGA, PLGA@SV and pmPLGA@SV. (e) Polydispersity index (PDI) of PLGA, PLGA@SV, and pmPLGA@SV. (f) Drug loading (DL%) and (g) entrapment efficiency (EE%) of SV in PLGA@SV and pmPLGA@SV. (h) In vitro cumulative release profile of SV from pmPLGA@SV in PBS (pH 7.4, 0.1% Tween 80, 37℃). (i) SDS-PAGE analysis of protein compositions from platelet membrane (lane 1) and pmPLGA@SV (lane 2). Numbers above bars indicate p-values. All quantitative data are presented as mean ± SD from three independent nanoparticle batches (n = 3).

The physicochemical properties of the synthesized nanoparticles were systematically characterized. TEM images revealed that PLGA, PLGA@SV, and pmPLGA@SV all exhibited a uniform spherical morphology (Fig 1a). Notably, pmPLGA@SV displayed a distinct core-shell structure, with a thin membrane layer successfully encapsulated around the PLGA core (Fig 1a, magnified view). DLS analysis showed that the hydrodynamic diameters of PLGA and PLGA@SV were approximately 150 nm, whereas the size of pmPLGA@SV approximately increased significantly to 165 nm (*p* = 0.0110), consistent with the addition of the membrane coating (Figs 1b and 1c, S1 Table). Zeta potential shifted from −32.2 mV (PLGA@SV) to −22.9 mV after platelet membrane coating (Fig 1d, S1 Table). The short-term stability of pmPLGA@SV was assessed at 4℃ in PBS (pH 7.4). The polydispersity index (PDI) values for PLGA, PLGA@SV, and pmPLGA@SV were all below 0.3, indicating narrow size distribution for all formulations (Fig 1e, S1 Table).

Furthermore, the EE and DL of SV in both PLGA@SV and pmPLGA@SV were assessed. Both formulations exhibited high EE (~65%) and DL (~6.2%), with no significant differences observed between the two groups, indicating that the membrane coating process did not induce premature drug leakage (Figs 1f and 1g). The stability of pmPLGA@SV was evaluated at 4℃ in PBS (pH 7.4). The encapsulation efficiency remained stable over 24 h with less than 10% drug leakage, and the hydrodynamic diameter showed no significant change (Supplementary S1 and S2 Figs). The in vitro release profile of SV from pmPLGA@SV exhibited an initial burst release (~18% within 24 h) followed by a sustained release phase (Fig 1h), indicating a stable and controlled release behavior of the nanoparticles.

To verify the successful translocation of platelet membrane proteins onto the nanoparticles, SDS-PAGE was performed (Supplementary S3 Fig). The protein profile of pmPLGA@SV closely matched that of the platelet membrane, confirming that the essential membrane proteins were successfully retained on the surface of the nanoparticles (Fig 1i).

### Enhanced cellular uptake and trans-BBB transport of pmPLGA nanoparticles

**Fig 2:**
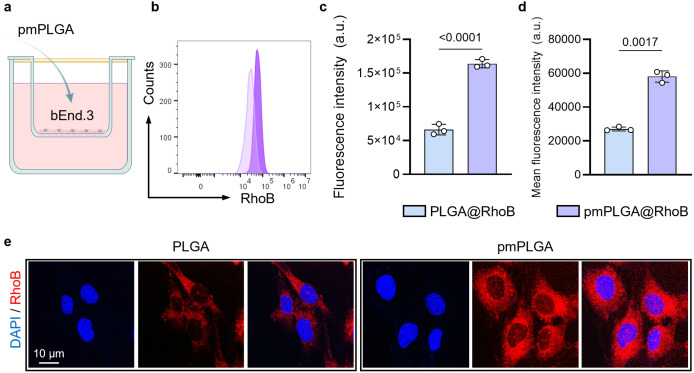
Cellular uptake and trans-barrier transport of biomimetic nanoparticles. (a) Schematic of the BBB model and the transport assay protocol. (b, c) Flow cytometry histograms and quantitative analysis of RhoB uptake in bEnd.3 cells (n = 3). (d) Fluorescence intensity of RhoB in the basolateral chamber, representing the trans-barrier efficiency across the OGD/R-treated bEnd.3 monolayer (n = 3). (e) Representative CLSM images of bEnd.3 cells showing the intracellular distribution of RhoB-labeled nanoparticles (red) and DAPI-stained nuclei (blue) (Representative images from three independent experiments (n = 3)). Numbers above bars indicate *p*-values. All measurements were performed using three independent batches (n = 3). Data are presented as mean ± SD.

To further investigate the delivery efficiency under pathological conditions, an in vitro ischemic BBB model was established using OGD/R-treated bEnd.3 monolayers (Fig 2a). Rhodamine B (RhoB) was encapsulated into the nanoparticles as a fluorescent tracer. Cellular uptake analysis via flow cytometry first confirmed that pmPLGA@RhoB significantly increased internalization by bEnd.3 cells compared to PLGA@RhoB (Fig 2b). The mean fluorescence intensity (MFI) of the pmPLGA@RhoB group was approximately 2.48-fold higher than that of the PLGA@RhoB group (*p* < 0.0001, Fig 2c). Crucially, the trans-barrier transport capability was quantified by measuring the fluorescence intensity in the basolateral chamber. In the OGD/R-injured model, pmPLGA@RhoB exhibited a significantly higher translocation efficiency than that of the PLGA@RhoB group (*p* < 0.0017, Fig 2d). This result indicates that the platelet membrane coating not only enhances cellular affinity but also facilitates the efficient penetration of nanoparticles across the compromised BBB. Complementary confocal laser scanning microscopy (CLSM) imaging further visualized the robust accumulation of pmPLGA@RhoB within the endothelial layer (Fig 2e), collectively demonstrating the superior brain-targeting potential of the biomimetic formulation under stroke-like conditions.

### Regulation of microglial polarization and neuroprotection against oxidative stress by pmPLGA@SV

**Fig 3:**
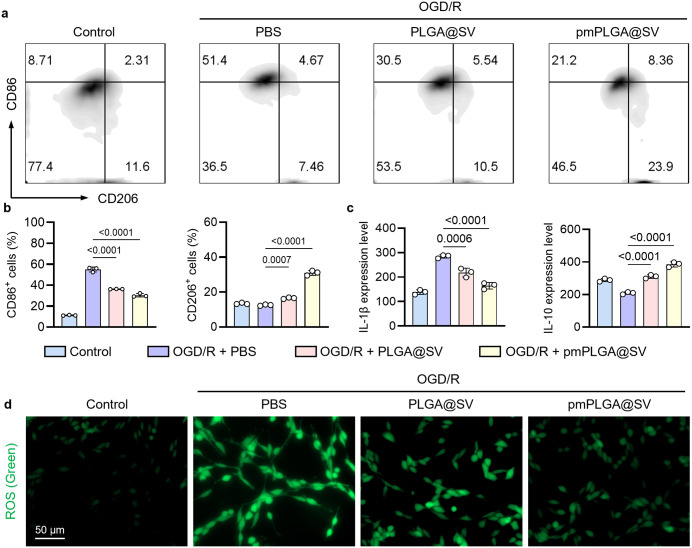
pmPLGA@SV modulates microglial polarization and alleviates neuronal oxidative stress post-OGD/R. (a) Representative flow cytometry scatter plots and (b) statistical analysis of M1 (CD86^+^) and M2 (CD206⁺) marker expression in BV2 microglia (n = 3). (c) Levels of pro-inflammatory (IL-1β) and anti-inflammatory (IL-10) cytokines in BV2 cells across different treatment groups (n = 3). (d) Representative fluorescence images showing intracellular ROS levels (green) in PC12 cells after various treatments (Representative images from three independent experiments (n = 3)). Numbers above bars indicate *p*-values. All measurements were performed using three independent batches (n = 3). Data are presented as mean ± SD.

To evaluate the multifaceted therapeutic effects of the biomimetic nanoparticles, we investigated their impact on microglial polarization and neuronal oxidative stress under OGD/R conditions (Supplementary S4 Fig). First, the immunomodulatory effect of pmPLGA@SV on BV2 cells were assessed via flow cytometry (Fig 3a). OGD/R insult significantly polarized BV2 cells toward a pro-inflammatory M1 phenotype, with the percentage of CD86 ⁺ cells reaching 56.07% (Fig 3b). However, treatment with pmPLGA@SV effectively reversed this trend, significantly reducing M1 markers while promoting the transition to an anti-inflammatory M2 phenotype (CD206⁺). This phenotypic switch was further confirmed by the cytokine profiles. pmPLGA@SV treatment markedly downregulated the expression of the pro-inflammatory cytokine IL-1β and upregulated the anti-inflammatory cytokine IL-10 in BV2 cells (Fig 3c), demonstrating a superior capacity to remodel the inflammatory microenvironment compared to the bare PLGA@SV group.

Beyond its anti-inflammatory role, we further explored the direct neuroprotective potential of pmPLGA@SV by monitoring ROS levels in PC12 cells. As shown in Fig 3d, OGD/R treatment induced a massive accumulation of intracellular ROS. Notably, the administration of pmPLGA@SV significantly attenuated the ROS-associated fluorescence, indicating its potent antioxidant capability in protecting neuronal-like cells from oxidative damage. Collectively, these findings suggest that pmPLGA@SV exerts a dual-functional therapeutic effect by simultaneously suppressing microglial-mediated neuroinflammation and mitigating neuronal oxidative stress.

### In vivo neuroprotective efficacy of pmPLGA@SV in tMCAO Mice

**Fig 4:**
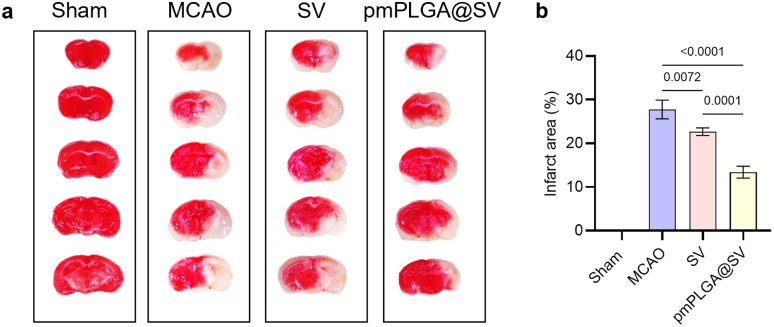
In vivo neuroprotective effects of pmPLGA@SV in tMCAO mouse model. (a) Representative images of TTC-stained coronal brain sections from the Sham, MCAO, SV, and pmPLGA@SV groups. The white areas represent the infarcted brain tissue (Representative images from three independent experiments (n = 3)). (b) Quantitative analysis of the infarct area as a percentage of the total brain volume (n = 3). Numbers above bars indicate *p*-values. All measurements were performed using three independent batches (n = 3). Data are presented as mean ± SD.

To evaluate the in vivo therapeutic potential of the biomimetic nanoparticles, we established a tMCAO mouse model. Brain infarct volumes were visualized using TTC staining 24 hours post-reperfusion (Fig 4a). As expected, no infarct was observed in the Sham group. In contrast, the MCAO group exhibited a substantial and well-defined infarct area (white region), with an average infarct percentage of approximately 27.8% (Fig 4b). Treatment with free SV significantly reduced the infarct area compared to the MCAO group (*p* = 0.0072). Notably, pmPLGA@SV treatment demonstrated the most pronounced neuroprotective effect, dramatically shrinking the infarct area to approximately 13.2% (*p* < 0.0001, vs. MCAO group). The therapeutic efficacy of pmPLGA@SV was also significantly superior to that of free SV (*p* = 0.0001), indicating that the platelet membrane coating remarkably enhances the brain-targeted delivery and bio-availability of SV. These results provide robust evidence that the pmPLGA@SV nano-system can effectively mitigate ischemic brain injury and preserve neural tissue integrity.

### Long-term functional recovery and neuro-rehabilitation facilitated by pmPLGA@SV

**Fig 5:**
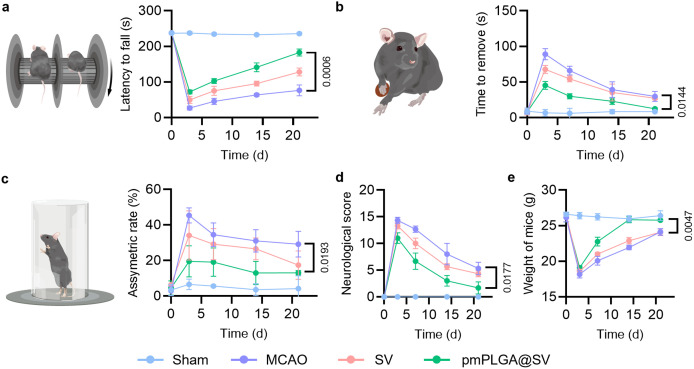
Long-term neurological functional recovery in tMCAO mice. (a) Motor coordination assessment via the rotarod test (n = 3). (b) Sensory-motor integration evaluation via the adhesive removal test (n = 3). (c) Spontaneous forelimb use asymmetry assessed by the cylinder test (n = 3). (d) Longitudinal neurological deficit scores following tMCAO (n = 3). (e) Body weight changes recorded over the 21-day observation period (n = 3). Numbers above bars indicate *p*-values. Data are presented as mean ± SD. Statistical significance is indicated only for the comparison between pmPLGA@SV and MCAO groups at day 21.

To further evaluate the impact of pmPLGA@SV on long-term neurological recovery, we performed a series of behavioral assessments over a 21-day period following MCAO injury. Initially, the rotarod test was employed to assess motor coordination and balance (Fig 5a). The MCAO group exhibited a sharp decline in the latency to fall compared to the Sham group. While free SV treatment led to moderate improvements, mice treated with pmPLGA@SV showed a significantly faster and more robust recovery of motor function throughout the observation period, with the latency to fall approaching Sham levels by day 21. Sensory-motor integration was evaluated using the adhesive removal test (Fig 5b). Post-tMCAO, mice required significantly more time to remove the adhesive tape. However, treatment with pmPLGA@SV markedly reduced this removal time, outperforming the free SV group and suggesting accelerated sensory-motor rehabilitation. Similarly, the cylinder test revealed a high asymmetric rate in the MCAO group, indicating impaired spontaneous forelimb use (Fig 5c). Administration of pmPLGA@SV effectively mitigated this lateralization, as evidenced by a consistent decrease in the asymmetric rate compared to both MCAO and SV groups.

The mNSS further corroborated these findings (Fig 5d). The pmPLGA@SV group demonstrated a steady and significant decline in deficit scores, indicating a continuous improvement in overall neurological status. Furthermore, the recovery of body weight (Fig 5e), which serves as an indicator of general health and vitality, was most prominent in the pmPLGA@SV group, with their weights nearly returning to baseline by the end of the study. Collectively, these results demonstrate that the biomimetic pmPLGA@SV nano-system significantly enhances long-term functional recovery and promotes neuro-rehabilitation following ischemic stroke.

## Discussion

Ischemic stroke triggers a devastating cascade of events, where the neurovascular unit (NVU) is compromised by oxidative stress, BBB breakdown, and persistent neuroinflammation [12,32]. In this study, we successfully engineered a platelet membrane–biomimetic nanoparticle system, pmPLGA@SV, for the targeted delivery of SV to the ischemic brain. Our findings indicate that pmPLGA@SV not only effectively reduces acute infarct volume but also significantly promotes long-term neurological recovery over a 21-day period. The observed therapeutic efficacy is associated with a synergistic mechanism that likely involves the active homing of platelet membranes to injured vasculature, the ROS-scavenging capability of SV, and the shift of microglial polarization toward a pro-healing M2 phenotype [12,14,15].

A major hurdle in stroke therapy is the efficient delivery of neuroprotective agents across the BBB to the ischemic penumbra. Conventional nanocarriers often suffer from rapid immune clearance and low targeting precision [33]. Our results suggest that pmPLGA@SV exhibits superior lesion-specific accumulation compared to non-coated nanoparticles. This enhanced targeting is probably mediated by the rich repertoire of surface proteins on the platelet membrane, which possess a natural affinity for activated endothelial cells and inflammatory sites within the cerebral vasculature [34]. By cloaking the PLGA core with this bio-interface, pmPLGA@SV may bypass systemic immune surveillance and concentrate SV at the lesion site, thereby maximizing its pleiotropic effects while minimizing systemic toxicity. However, it should be noted that we did not perform competitive inhibition experiments (e.g., using blocking antibodies against GPVI or P-selectin) to directly demonstrate receptor-mediated uptake or BBB transcytosis. Such mechanistic studies are warranted in future work to dissect the precise molecular pathways involved in the active homing of this biomimetic platform.

The interplay between oxidative stress and neuroinflammation is a hallmark of ischemia–reperfusion injury [35]. In our in vitro studies using PC12 and BV2 cells, pmPLGA@SV demonstrated a dual-functional capacity to remodel the pathological microenvironment. The significant reduction in ROS levels suggests that SV, when delivered via this targeted platform, exerts neuroprotective effects by attenuating neuronal oxidative stress. More importantly, we observed a repolarization of microglia from the pro-inflammatory M1 phenotype toward the anti-inflammatory M2 phenotype. This shift is considered beneficial, as M2 microglia secrete neurotrophic factors and anti-inflammatory cytokines (e.g., IL-10), which can mitigate secondary inflammatory damage and provide a supportive niche for surviving neurons [36]. Nevertheless, the precise molecular mechanisms linking SV delivery to microglial phenotypic switching remain to be elucidated; pathway-specific inhibitors or genetic models are required to establish causality (e.g., involvement of NF-κB, STAT3, or Nrf2 pathways).

A distinctive highlight of this research is the comprehensive evaluation of long-term functional recovery. While many studies focus solely on acute-phase survival (1–7 days), our 21-day observation period provides more clinically relevant evidence of neuro-rehabilitation [37]. The consistent improvements in motor coordination (rotarod), sensory-motor integration (adhesive removal), and spontaneous forelimb symmetry (cylinder test) collectively suggest that pmPLGA@SV facilitates sustained functional restoration, not merely transient protection. The observed correlation between reduced infarct volume and long-term behavioral recovery supports the premise that attenuating neuronal oxidative stress and modulating the inflammatory response during the acute phase may translate into sustained neurological improvements [38].

Several limitations of this study should be explicitly acknowledged. First, we did not perform formal pharmacokinetic (PK) studies or quantitative biodistribution analysis (e.g., tissue accumulation of pmPLGA@SV in brain, liver, spleen). Although our in vitro trans-BBB model and in vivo therapeutic efficacy strongly suggest lesion-targeted delivery, direct evidence of the nanoparticle’s distribution profile and circulation half-life is lacking. Future work will include fluorescence imaging and PK analysis. Second, the behavioral studies were conducted with a modest sample size (n = 3 per group). While the effect sizes were large and consistent across animals, resulting in low variability, a larger cohort (e.g., n ≥ 8 per group) is required for confirmatory studies to ensure generalizability. Third, only male mice were used; future studies should evaluate efficacy in female mice to assess potential sex-dependent differences. Fourth, competitive inhibition experiments (e.g., using blocking antibodies against GPVI or P-selectin) were not performed to directly confirm receptor‑mediated uptake or BBB transcytosis, which remains a key direction for future mechanistic studies. Finally, the precise downstream signaling cascades mediating the pleiotropic effects of pmPLGA@SV remain to be directly tested.

## Conclusions

In conclusion, the pmPLGA@SV biomimetic system represents a promising platform with further scope of validation for ischemic stroke treatment. By integrating the targeting capabilities of platelet membranes with the multi-targeted therapeutic potential of simvastatin, we achieved a synergistic effect that addresses the complexity of stroke pathology. Future studies will focus on clinical translation, including the scalability of the membrane-coating process and the evaluation of this platform in large animal models and both sexes. Nevertheless, our findings provide a compelling proof-of-concept for using biomimetic nanotechnology to bridge the gap between drug discovery and effective clinical neuroprotection.

## Supporting information

S1 Fig Drug loading retention of nanoparticles during storage at 4℃ within 14 days. (TIF)

S2 Fig Size stability of nanoparticles measured as hydrodynamic diameter over a 14-day storage period at 4℃. (TIF)

S3 Fig Original uncropped SDS-PAGE analysis of protein compositions from the platelet membrane and the membrane-coated nanoparticles (pmPLGA@SV). (JPG)

S4 Fig Representative flow cytometry gating strategy. Live cells were gated on FSC-A/SSC-A, doublets excluded by FSC-A/FSC-H and FSC-H/FSC-W, and then CD206 vs. CD86 analyzed on singlet population. Quadrant gates based on control sample.
(TIF)

S1 Table Physicochemical characterization of PLGA, PLGA@SV, and pmPLGA@SV nanoparticles (size, zeta potential, PDI). (DOCX)
