# Supplementary figures and images for: Platelet membrane biomimetic nanoparticle-based targeted delivery system of simvastatin for the treatment of ischemic stroke
Source: PLoS One. 2026 Jul 23;21(7):e0354184. doi: 10.1371/journal.pone.0354184 (PMC13395312; doi:10.1371/journal.pone.0354184)

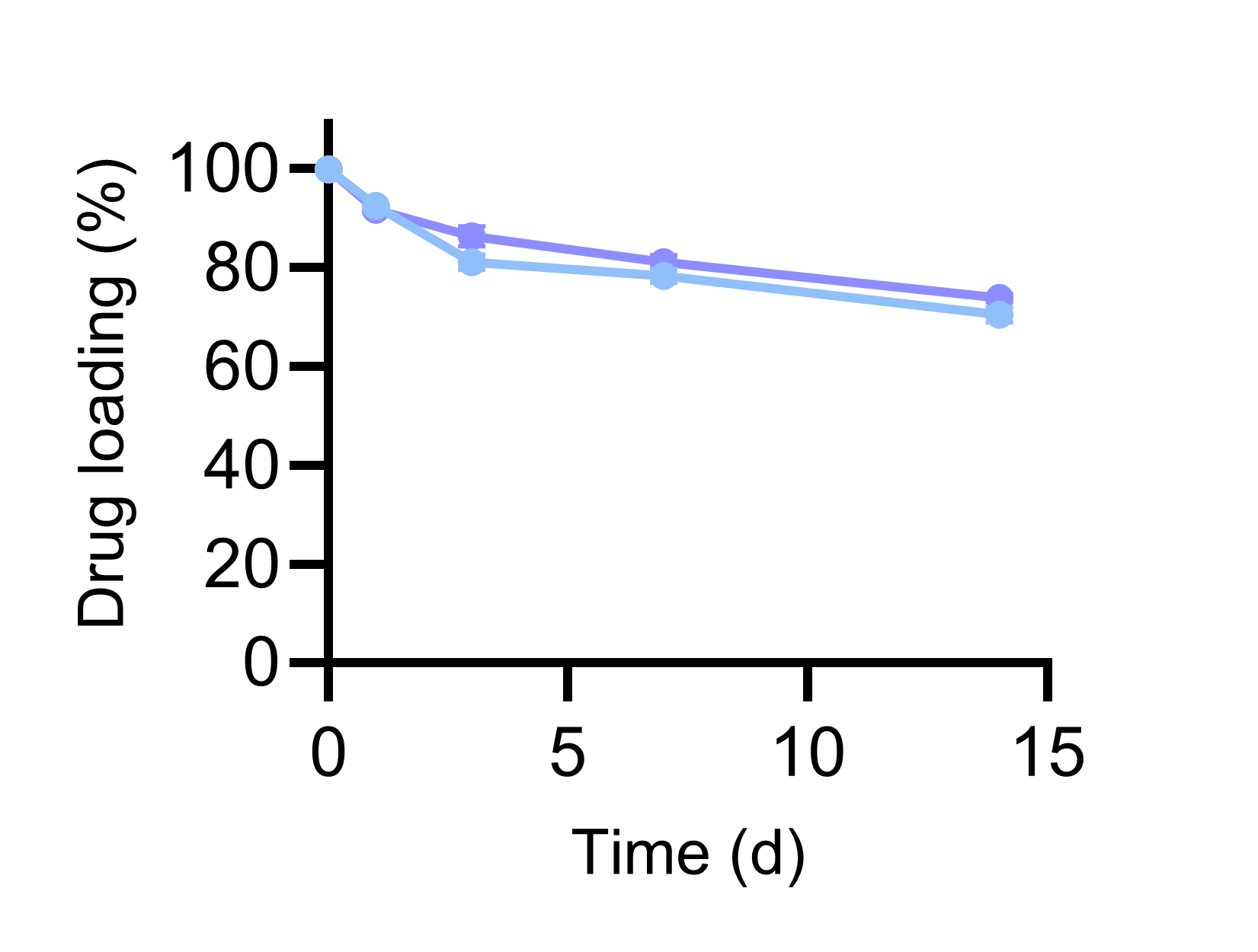

Supplement: S1 Fig — (TIF) [file pone.0354184.s001.tif]

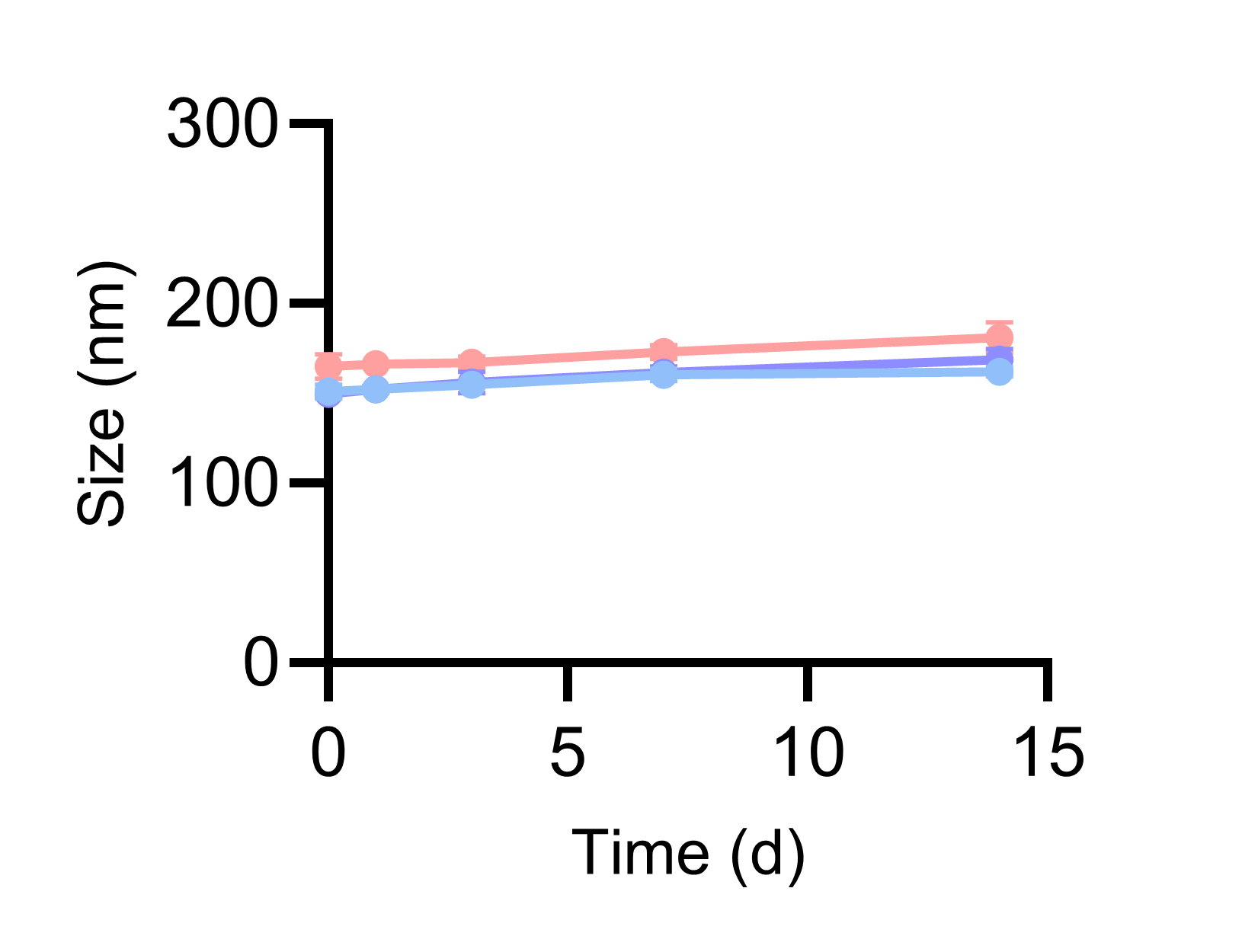

Supplement: S2 Fig — (TIF) [file pone.0354184.s002.tif]

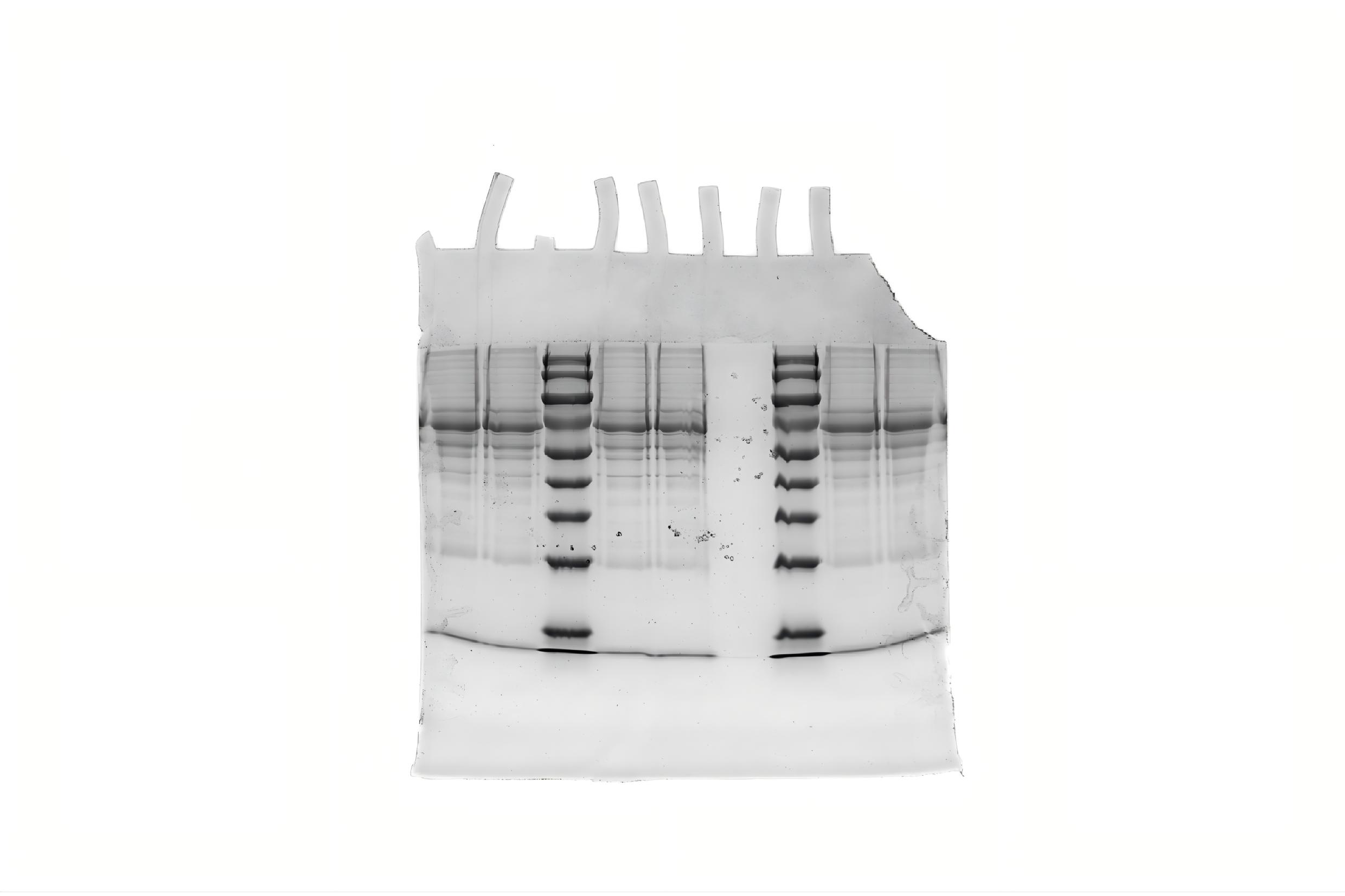

Supplement: S3 Fig — (JPG) [file pone.0354184.s003.jpg]

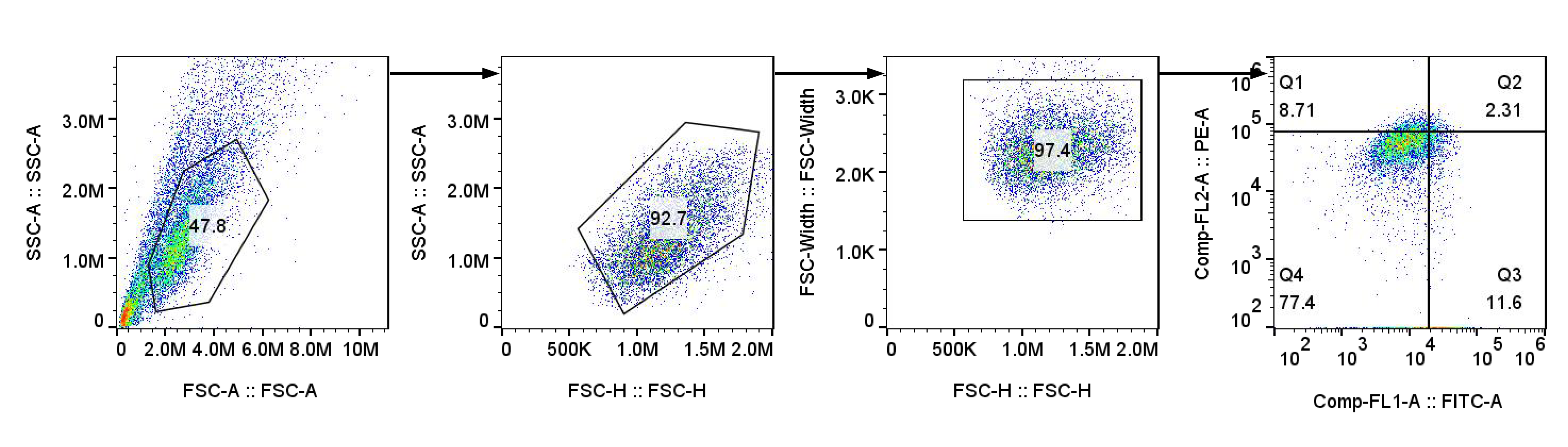

Supplement: S4 Fig — Live cells were gated on FSC-A/SSC-A, doublets excluded by FSC-A/FSC-H and FSC-H/FSC-W, and then CD206 vs. CD86 analyzed on singlet population. Quadrant gates based on control sample. (TIF) [file pone.0354184.s004.tif]
