# Supplementary material for: Platelet membrane biomimetic nanoparticle-based targeted delivery system of simvastatin for the treatment of ischemic stroke
Source: PLoS One. 2026 Jul 23;21(7):e0354184. doi: 10.1371/journal.pone.0354184 (PMC13395312; doi:10.1371/journal.pone.0354184)
Supplement: S1 Table — (DOCX) [file pone.0354184.s005.docx]

**Table S1. Physicochemical characterization of PLGA, PLGA@SV, and pmPLGA@SV nanoparticles (size, zeta potential, PDI).**

| Sample | Size (nm) | Zeta potential (mV) | PDI | n |
| --- | --- | --- | --- | --- |
| PLGA | 151.73 ± 6.06 | -31.73 ± 2.07 | 0.19 ± 0.01 | 3 |
| PLGA@SV | 152.78 ± 4.54 | -32.23 ± 3.37 | 0.20 ± 0.01 | 3 |
| pmPLGA@SV | 166.53 ± 0.78 | -22.90 ± 1.47 | 0.22 ± 0.01 | 3 |
